# Supplementary material for: Validation of a spring loaded probe for single and repeat pressure pain testing, including public domain specifications for design and manufacture
Source: PLoS One. 2024 Jan 12;19(1):e0292809. doi: 10.1371/journal.pone.0292809 (PMC10794070; doi:10.1371/journal.pone.0292809)
Supplement: S1 File — (DOCX) [file pone.0292809.s001.docx]

Supporting information

## Supporting information

Linear mixed model fit by REML. t-tests use Satterthwaite's method [
lmerModLmerTest]
Formula: value ~ tester + day + rep_number + test_site + tool + session +
 (1 | id)
 Data: LMM_data

REML criterion at convergence: 3104.1

Scaled residuals:
 Min 1Q Median 3Q Max
-3.5578 -0.6314 -0.0333 0.6332 3.0335

Random effects:
 Groups Name Variance Std.Dev.
 id (Intercept) 2.579 1.606
 Residual 1.863 1.365
Number of obs: 864, groups: id, 27

Fixed effects:
 Estimate Std. Error df t value Pr(>|t|)
(Intercept) 6.85305 0.35274 40.80047 19.428 < 2e-16 ***
testerX 0.02318 0.12372 834.98399 0.187 0.8514
testerY -0.52319 0.24185 822.14294 -2.163 0.0308 *
testerZ -0.22701 0.22853 820.39574 -0.993 0.3208
dayB 0.10290 0.09338 828.98961 1.102 0.2708
rep_numberb -0.46137 0.09287 828.83102 -4.968 8.22e-07 ***
test_siteLower leg -1.28016 0.09287 828.83102 -13.785 < 2e-16 ***
toolPressure probe 1.25488 0.09287 828.83102 13.513 < 2e-16 ***
sessionb -0.19618 0.09928 841.93730 -1.976 0.0485 *
---
Signif. codes: 0 '***' 0.001 '**' 0.01 '*' 0.05 '.' 0.1 ' ' 1

Correlation of Fixed Effects:
 (Intr) testrX testrY testrZ dayB rp_nmb tst_Ll tlPrsp
testerX -0.240
testerY -0.322 0.403
testerZ -0.331 0.439 0.792
dayB -0.132 0.055 -0.042 0.009
rep_numberb -0.132 0.000 0.000 0.000 0.000
tst_stLwrlg -0.132 0.000 0.000 0.000 0.000 0.000
tolPrssrprb -0.132 0.000 0.000 0.000 0.000 0.000 0.000
sessionb -0.225 0.049 0.337 0.285 -0.019 0.000 0.000 0.000

Mixed linear regression model on pressure pain threshold. As fixed effects, we entered tool, test_site, rep_number, tester, day and session (without interaction term) into the model. As random effects, we had intercepts for subjects.

Model: value ~ tester + day + rep_number + test_site + tool + session + (1 | id). The random effects table shows that the individuals intercepts vary with a SD of 1.61 and the SD of error not accounted for by individual is 1.37.

Furthermore pairwise t-tests shows that there are a statistically significant in the fixed effects of tester, repetition number, anatomical site, tool and session.

| 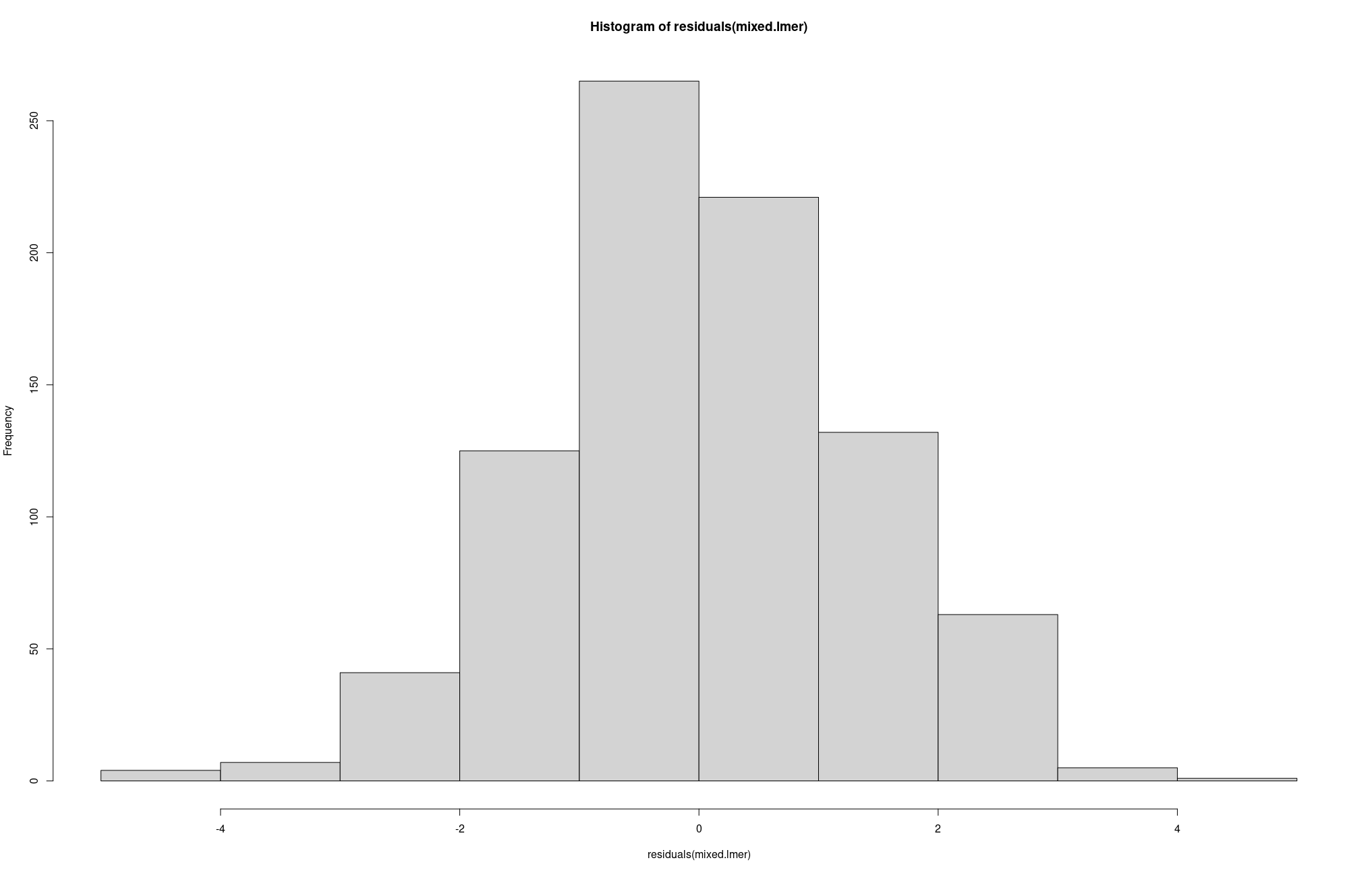  Histogram of the residuals of the mixed linear regression model showing that assumptions of normality hold. |
| --- |

| 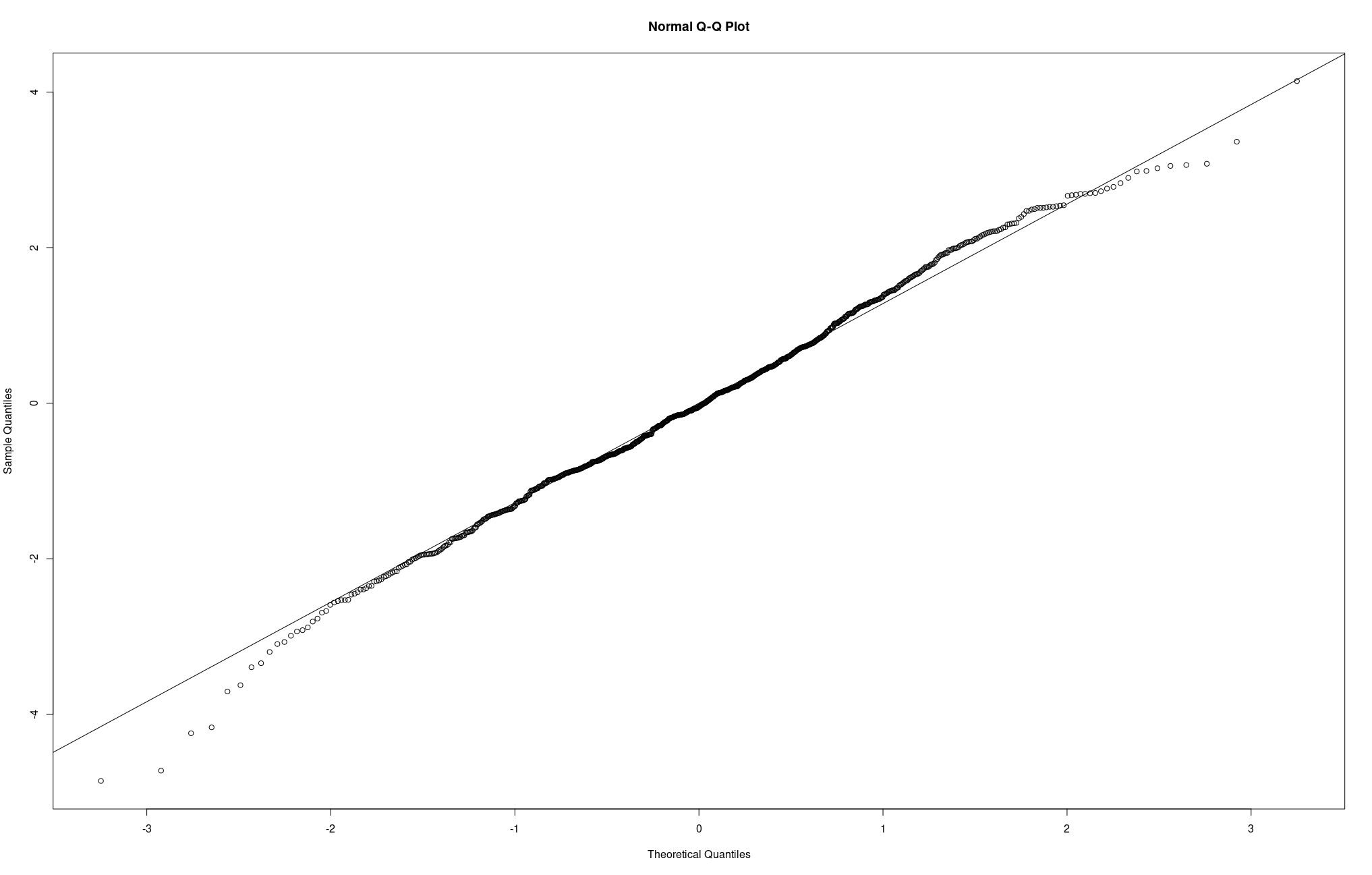  Q-Q plot indicates that the data falls on a straight line, which indicates that there are no obvious violation of the normality assumption. |
| --- |
