# Supplementary material for: Validation of a spring loaded probe for single and repeat pressure pain testing, including public domain specifications for design and manufacture
Source: PLoS One. 2024 Jan 12;19(1):e0292809. doi: 10.1371/journal.pone.0292809 (PMC10794070; doi:10.1371/journal.pone.0292809)
Supplement: Attachment — Submitted filename: Response_to_reviewers_2.pdf [file pone.0292809.s004.pdf]

Response to Reviewers 2

S. O'Neill

Dear editor and reviewers

Thank you very much for a second review of our manuscript, which has further improved it, in our view.

Review comments to the author

Reviewer #1

We thank the reviewer for their efforts and for recommending our manuscript for publication.

Reviewer #2

We thank the reviewer for additional comments, which we address below:

- To increase the accuracy of the expression, the paragraph

We have amended the first paragraph of *Discussion/Force measurement* as suggested by reviewer #2. It now reads:

> The force measurement platform was programmed to read sensor data continuously rather than enforcing a fixed frequency of sampling, which resulted in a small degree of variation in sampling rates. This is unlikely to make any difference in the current results, but for future implementation it could be useful to **upgrade the acquisition software in order to synchronize sampling with an accurate time base generated by a hardware timer (present inside the ATmega328 processor).**

Text highlighted in bold, has been revised.

- Before equation 2 it could be useful to change the

We have amended the relevant sentence in *Methods and materials/Pressure probe test setup/Time series data analyses* as suggested by reviewer #2. It now reads:

> Equation 2 **describes the way to obtain the optimal warping path by minimization of the the Euclidean distance.**

Text highlighted in bold, has been revised.

- In Discussion section at "Rate of pressure application" paragraph it could be useful to add a comment about the results presented in Figure 13 and Table 3 indicating that the operator needs sufficient instruction to actually understand the task at hand.

We agree and thank the reviewer for pointing this out. That point was, to some extent, made in the preceding section, but we agree it bears being made explicit in the "Rate of pressure application" section also. We have added the following to that section:

> **The small number of testers who, despite an audio cue, had a rate of application twice that of the target rate, illustrates the need for sufficient instruction of testers.**
>
> We recommend the use of a sound cue to ensure that testers can reliably maintain the correct rate of pressure application with little variation **and we recommend that instructions in the use of pressure probes, be supplemented by practice sessions supervised by an experienced tester.**

Text highlighted in bold, has been added

We thank the reviewer for recommending to accept our manuscript after these minor revisions.

On behalf of the authors

S. O'Neill – Sep 20th 2023
