# Supplementary material for: Validation of a spring loaded probe for single and repeat pressure pain testing, including public domain specifications for design and manufacture
Source: PLoS One. 2024 Jan 12;19(1):e0292809. doi: 10.1371/journal.pone.0292809 (PMC10794070; doi:10.1371/journal.pone.0292809)
Supplement: Attachment — Submitted filename: PONE-D-23-13550_comments.docx [file pone.0292809.s003.docx]

An interesting study on validation of a simple mechanical test apparatus to assess the temporal summation of deep pressure pain. Therefore, temporal summation and pressure pain are two key measuring issues of this study. The authors initiated as “temporal summation of pressure pain is technically more challenging than simple pressure pain thresholds”. However, simple relationship or contrast of temporal summation pain (TSP) and pressure pain threshold (PPT) are not established from scientific literature in this manuscript. The following articles may assist the authors to contrast TSP and PPT in clinical scenario:

1. A study (https://pubmed.ncbi.nlm.nih.gov/33316140/) indicated a clinical presentation with psychological factors (one dimension of risk) is not associated with high-PPT/sensitivity (another dimension of risk).

2. The authors can also improve discussion by taking evidence from this paper ( https://pubmed.ncbi.nlm.nih.gov/30648781/) by indicating the differential predictive value of dynamic (TSP) versus static (PPT) measures of pain sensitivity as well as the unique value of evoked measures (and the potential limitations of psychological factors) in predicting functional outcome.

3. A commentary article, available at <https://journals.lww.com/clinicalpain/Citation/2021/04000/Movement_evoked_Pain__MEP_.8.aspx> , may help authors making a nice future direction on usefulness of QST in MSK pain mechanism including temporal summation of pressure pain.

I hope after a revision reader will be more clear on science link clinical application of TSP and PPT as well as this new evolving technique of this manuscript (temporal summation of pressure pain).
